# Isoprenoids from the Soft Coral *Sarcophyton glaucum*

**DOI:** 10.3390/md15070202

**Published:** 2017-06-27

**Authors:** Chih-Hua Chao, Wen-Liang Li, Chiung-Yao Huang, Atallah F. Ahmed, Chang-Feng Dai, Yang-Chang Wu, Mei-Chin Lu, Chih-Chuang Liaw, Jyh-Horng Sheu

**Affiliations:** 1 School of Pharmacy, China Medical University, Taichung 404, Taiwan; chchao@mail.cmu.edu.tw; 2 Chinese Medicine Research and Development Center, China Medical University Hospital, Taichung 404, Taiwan; 3 Department of Marine Biotechnology and Resources, National Sun Yat-sen University, Kaohsiung 804, Taiwan; m025020018@student.nsysu.edu.tw (W.-L.L.); huangcy@mail.nsysu.edu.tw (C.-Y.H.); ccliaw@mail.nsysu.edu.tw (C.-C.L.); 4 Department of Pharmacognosy, College of Pharmacy, King Saud University, Riyadh 11451, Saudi Arabia; afahmed@ksu.edu.sa; 5 Institute of Oceanography, National Taiwan University, Taipei 112, Taiwan; corallab@ntu.edu.tw; 6 Graduate Institute of Natural Products, Kaohsiung Medical University, Kaohsiung 807, Taiwan; yachwu@mail.cmu.edu.tw; 7 Research Center for Natural Products & Drug Development, Kaohsiung Medical University, Kaohsiung 807, Taiwan; 8 Department of Medical Research, Kaohsiung Medical University Hospital, Kaohsiung 807, Taiwan; 9 Graduate Institute of Marine Biotechnology, National Dong Hwa University, Pingtung 944, Taiwan; jinx6609@nmmba.gov.tw; 10 National Museum of Marine Biology & Aquarium, Pingtung 944, Taiwan; 11 Department of Medical Research, China Medical University Hospital, China Medical University, Taichung 404, Taiwan; 12 Frontier Center for Ocean Science and Technology, National Sun Yat-sen University, Kaohsiung 804, Taiwan

**Keywords:** *sarcophyton glaucum*, lobocrasol, *ent*-sarcophyolide E, sarcophine

## Abstract

Five new isoprenoids, 3,4,8,16-tetra-*epi*-lobocrasol (**1**), 1,15β-epoxy-deoxysarcophine (**2**), 3,4-dihydro-4α,7β,8α-trihydroxy-Δ^2^-sarcophine (**3**), *ent*-sarcophyolide E (**4**), and 16-deacetyl- halicrasterol B (**5**) and ten known compounds **6**‒**15**, were characterized from the marine soft coral *Sarcophyton glaucum*, collected off Taitung coastline. Their structures were defined by analyzing spectra data, especially 2D NMR and electronic circular dichroism (ECD). The structure of the known compound lobocrasol (**7**) was revised. Cytotoxicity potential of the isolated compounds was reported, too.

## 1. Introduction

Soft corals classified in the genus *Sarcophyton* are the predominant species in many coral reefs. They are endowed with diverse secondary metabolites, including cembranoids [1,2], biscembranoids [3] and steroids [4]. For pharmacological research, some of the metabolites are reported to possess cytotoxic [5,6], antimicrobial [7], and neuroprotective [8] activities. Ecologically, sarcophytoxide, usually found in sarocophyton species, is an allelopathic chemical used in competition for space with scleractinian corals [9]; while another well-known metabolite, sarcophine, is known to have a toxic effect on fishes [10]. In our previous work, we had isolated novel biscembranoids with cytotoxic activity and anti-inflammatory properties from the cultured soft coral *S. glaucum* [3]. Our present investigation disclosed the purification and structural elucidation of five new and ten known isoprenoid-derived compounds from the same wild-type species, collected off the coastline of Taitung County. The structure of **7** was also revised. Cytotoxic activity of the isolates was assayed by the inhibition of cancer cell proliferation.

## 2. Results and Discussion

**Figure 1:**
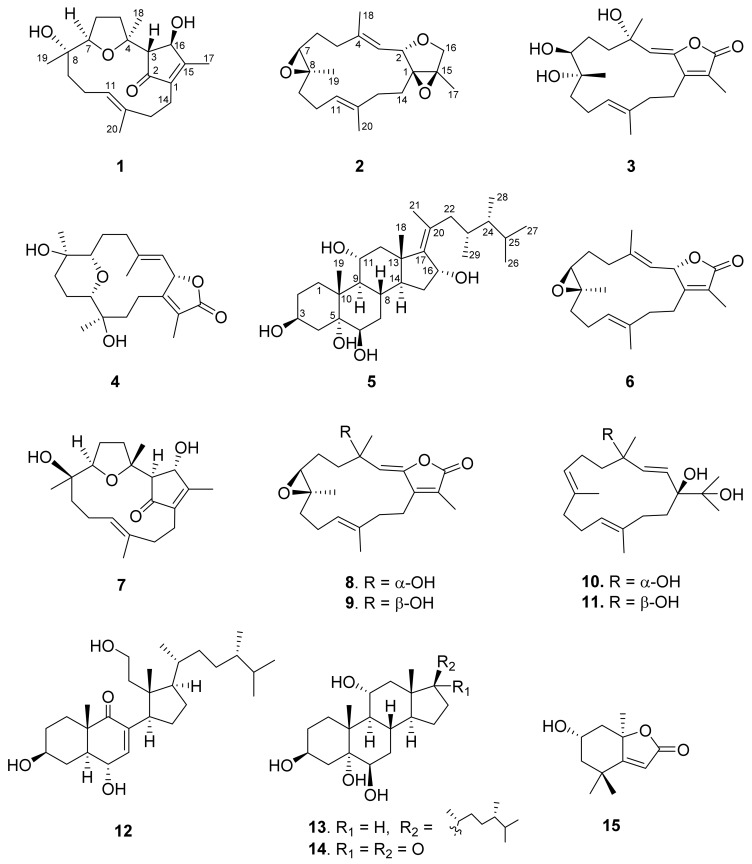
Structures of compounds **1**–**15**.

The EtOAc extract of *Sarcophyton glaucum* was repeatedly separated by column chromatography and HPLC to obtain five new isoprenoids **1**‒**5** and ten known compounds **6**‒**15** (Figure 1). By comparing with data in the literature, the known compounds were identified as sarcophine (**6**) [11], lobocrasol (**7**) [12], 3,4-dihydro-4α-hydroxy-Δ^2^-sarcophine (**8**) [11,13], 3,4-dihydro-4β-hydroxy-Δ^2^-sarcophine (**9**) [11,13], crassumol A (**10**) [14], klyflaccicembranol F (**11**) [15], sarcomilasterol (**12**) [16], sarcoaldesterol B (**13**) [17], sarglaucsterol (**14**) [18], loliolide (**15**) [19].

The molecular formula of 3,4,8,16-tetra-*epi*-lobocrasol (**1**), a colorless oil, was determined as C_20_H_30_O_4_ based on the [M + Na]^+^ ion peak obtained by (+)-HRESIMS. The ^13^C NMR data showed 20 carbon signals, including evidence of an α,β-unsaturated enone (*δ*_C_ 204.3, 167.0, and 142.8), an additional double bond (*δ*_C_ 133.3 and 128.4), and four oxygenated carbons (*δ*_C_ 83.8, 83.2, 75.9, and 73.6) (Table 1). The ^1^H NMR, in conjunction with the HSQC spectra, revealed that the structure of **1** possessed four methyl groups [*δ*_H_ 2.08 (3H, s), 1.69 (3H, s), 1.35 (3H, s), 1.03 (3H, s)], an olefinic methine [*δ*_H_ 5.15 (1H, t, *J* = 7.6 Hz)], and two oxygenated methines [*δ*_H_ 4.90 (1H, s); 3.80 (1H, dd, *J* = 9.2, 5.6 Hz)] (Table 2). The above spectral data were similar to those of lobocrasol (**7**) [12], suggesting that **1** might be an epimer of **7**.

**Figure 2:**
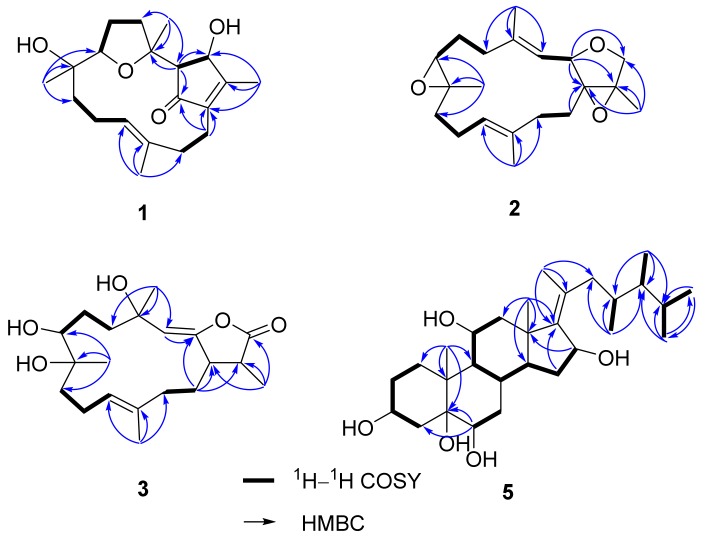
Selected ^1^H–^1^H COSY and HMBC correlations of **1**–**3** and **5**.

**Figure 3:**
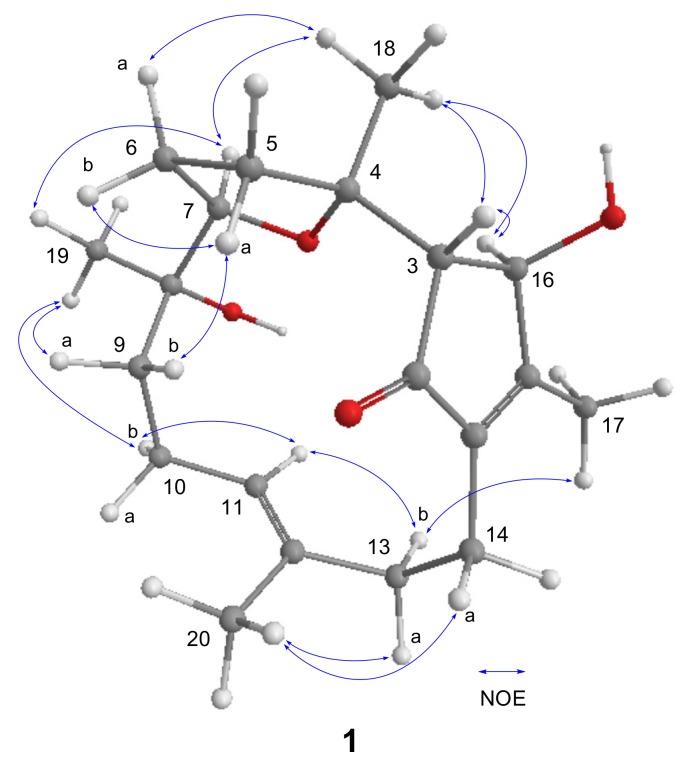
Selected NOE correlations of compound **1**.

**Figure 4:**
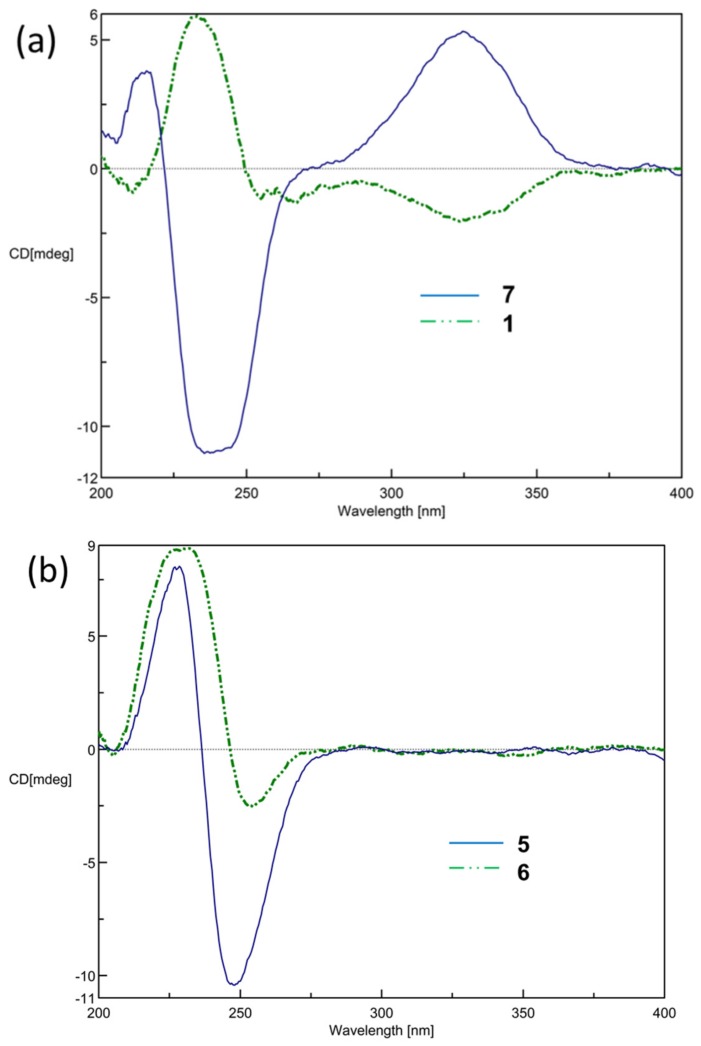
Electronic circular dichroism (ECD) curves of (**a**) compounds **1** and **7**; (**b**) compounds **5** and **6**.

Analysis of COSY spectra established four proton spin systems: H-3/H-16, H_2_-5/H_2_-6/H-7, H_2_-9/H_2_-10/H-11, and H_2_-13/H_2_-14 (Figure 2). The HMBC correlations from H_3_-18 to C-3, C-4, and C-5 made the connection of the tetrahydrofuran ring and the 4-hydroxy-3-methylcyclopent-2-enone moiety. The HMBC correlations from H_3_-19 to C-7, C-8, and C-9; from H_3_-20 to C-11, C-12, and C-13; and from H_3_-17 to C-1, C-15, and C-16 combined with correlations from H_2_-14 to C-1, C-2, and C-15 constructed the remaining fragments. Consequently, the planar structures of **1** and **7** were found to be the same. The relative configurations of all the chiral centers in **1** were deduced by interpretation of nuclear Overhauser effect (NOE) data, analysis of ^3^*J*_H-H_ values, and comparison of carbon chemical shifts. As depicted in Figure 3, the NOE cross-peaks of H_3_-18/H-7 supported that they oriented on the same face, and casually assigned these protons to be α-oriented. The downfield-shifted proton H-5a (*δ*_H_ 3.04), deshielded by the C-2 carbonyl group, showed an NOE correlation with H-9b (*δ*_H_ 1.25), while H_3_-19 showed correlations with both H-7 and H-9a (*δ*_H_ 1.50), suggesting that H-7 and C-9 were located in anti-orientation, thus suggesting a β-orientation for H_3_-19. The strong NOE cross-peaks of H_3_-18/H-16 suggested the α-orientation of H-16. A weak NOE correlation was observed between H-16 and H-3, while H-3 also showed a correlation with H_3_-18, and suggested H-3 should be located anti to H-16. This was confirmed by comparing the small ^3^*J*_H-H_ values with those of cyclopent-2-enone analogues, in which adjacent protons with a *trans* configuration showed small coupling constants [20,21]. The *E* geometry for Δ^11^ double bond was deduced by NOE cross-peaks of H-11/H-13b (*δ*_H_ 1.84), as well as by comparing carbon chemical shift for the C-20 at *δ*_C_ 15.3 (<20 ppm) [22]. Thus, the chemical shift for C-20 of **7** (*δ*_C_ 14.8) revealed the Δ^11^ double bond should possess the *E*, not *Z* geometry, as assigned in the literature [12]. This was further confirmed by our observation of NOE correlation between H-11 and H-13 (*δ*_H_ 2.48) (Supplementary Materials, Figure S6-3). The absolute configuration at C-16 of **7** was determined by Lin et al. using Mosher’s method [12]. The electronic circular dichroism (ECD) spectrum of **1** showed Cotton effects with approximately opposite signs compared to that of **7** (Figure 4a), suggesting different configurations at C-3 and C-16 for both **1** and **7**. Thus, the absolute configuration of **1** was determined as shown in Figure 1.

**Figure 5:**
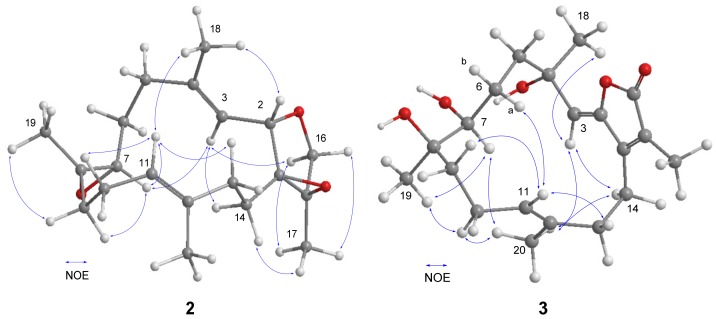
Selected NOE correlations of compounds **2** and **3**.

The 1,15β-Epoxy-deoxysarcophine (**2**) was isolated as a colorless oil and had a molecular formula of C_20_H_30_O_3_, determined by HRESIMS analysis. The NMR spectra were quite similar to those of 1,15β-epoxy-2-*epi*-deoxysarcophine [23] with the exception of the signals around the tetrahydrofuran (THF) ring. The ^1^H–^1^H COSY and HMBC correlations, as depicted in Figure 2, confirmed that **2** and 1,15β-epoxy-2-*epi*-deoxysarcophine shared the same planar structure. The 14-membered ring of **2** is suggested to be identical to that of sarcophine (**6**), of which the C-7 to C-11 fragment adopted a half-chair conformation [24], due to the fact that both compounds possessed similar key NOE correlation data: H-3/H-7 and H-7/H-11 (Figure 5). The NOE cross-peaks of H-3/H_2_-14, H_2_-14/H_3_-17, as well as H-2/H_3_-18, implied that both the 1,15-epoxy group and H-2 were β-oriented.

The molecular formula of 3,4-dihydro-4α,7β,8α-trihydroxy-Δ^2^-sarcophine (**3**), C_20_H_30_O_5_, was established by HRESIMS, exceeding that of **8** or **9** by 18 mass units. A series of characteristic absorption bands due to hydroxy (3444 cm^−1^) and carbonyl (1747 cm^−1^) groups were assigned from the IR spectrum of **3**. The NMR data of **3** resembled those of **8** and **9**, with significant differences in carbon chemical shifts at C-7 (*δ*_C_ 74.6) and C-8 (*δ*_C_ 74.9), suggesting that **3** is a 7,8-dihydroxy analogue of **8** or **9**. The Δ^2^ and Δ^11^ double bonds were both determined as *E* geometry according to NOE cross-peaks of H-3/H_2_-14 and H-10/H_3_-20, respectively (Figure 5). Moreover, the correlations of H-7/H_3_-19, H-7/H_3_-20, and H-3/H_3_-20 disclosed that these protons located on the same face and subjectively designated as α protons. The above correlations, as well as that of H-6a/H-11, restricted the envenlop conformation for the C-7 to C-11 segment (Figure 5). Accordingly, H_3_-18 was correlated with H-3, but not with H-7, suggesting that this methyl group is likely β-oriented.

*ent*-Sarcophyolide E (**4**), a colorless oil, was found to have a molecular formula of C_20_H_30_O_5_, based on its HRESIMS. Its NMR spectroscopic data (Table 1 and Table 2) were superimposed to that of sarcophyolide E [11]. The ECD spectra of **4** showed a negative Cotton effect for n→π* (254 nm) and positive π→π* (230 nm) transitions, which are quite the same as those of the co-isolated sarcophine (**6**) (Figure 4b), but are in opposite signs to those reported for sarcophyolide E [11]. Accordingly, **4** was elucidated as an enantiomer of sarcophyolide E. Surprisingly, the specific optical rotation of sarcophyolide E was reported as [α]_D_ +4, that has the same positive sign as the specific optical rotation value of **4** ([α]_D_ +14). The [α]_D_ of **4** was repeatedly measured and the positive sign was always obtained. The reason for this discrepancy with literature might need to be studied further.

The HRESIMS of 16-deacetyl-halicrasterol B (**5**) showed a sodiated adduct ion peak at *m*/*z* 501.3552, implying a molecular formula of C_29_H_50_O_5_. Its ^1^H NMR spectrum disclosed three methyl singlets (one assignable to be olefinic methyl), four methyl doublets, four oxygenated-methine protons [4.00 (m, H-3); 3.47 (br s, H-6); 3.88 (td, J = 10.4, 5.2 Hz); 4.62 (d, J = 5.2 Hz, H-16)], as well as a complex array of aliphatic methine and methylene protons (Table 3). The ^13^C NMR spectrum showed a tetra-substituted double bond at *δ*_C_ 148.2 (C-17) and 134.2 (C-20) and five oxygenated carbons at *δ*_C_ 68.2 (C-3), 77.4 (C-5), 76.6 (C-6), 69.6 (C-11), and 73.0 (C-16). The above spectroscopic data were very similar to those of a Δ^17(20)^ sterol, halicrasterol B, with the acetyl signals disappeared in **5**, suggesting that **5** is a deacetyl derivative of halicrasterol B. This was secured by analyzing the ^1^H–^1^H COSY correlations and the HMBC experiments (Figure 2). Compound **5** and halicrasterol B were found to share the same configurations at the side chain segment based on their identical ^13^C NMR data. Accordingly, the structure of **5** was characterized as depicted in Figure 1.

The cytotoxicity of the isolates **1**–**15** against HepG2 (human hepatocellular liver carcinoma), MDA-MB-231 (human breast adenocarcinoma), and A-549 (human lung epithelial cells) cancer cells were assayed. Furthermore, compounds **6**, **8**, **9**, and **12** were also assayed for cytotoxicity against MOLT-4 (human acute lymphoblastic leukemia), SUP-T1 (human T-cell lymphoblastic lymphoma), and U-937 (human histiocytic lymphoma) cell lines. The results showed that compound **12** exhibited cytotoxicity effect against MDA-MB-231, MOLT-4, SUP-T, and U-937 cell lines with IC_50_ values of 13.8, 6.7, 10.5, and 17.7 μg/mL, respectively, while compound **13** was found to possess cytotoxicity against HepG2, MDA-MB-231, and A-549 cell lines with IC_50_ values of 9.7, 14.0, and 15.8 μg/mL. The remaining compounds were found to be not cytotoxic against the above cancer cell lines, with IC_50_ values higher than 20 μg/mL.

## 3. Experimental Section

### 3.1. General Experimental Procedures

The optical rotation values and IR spectra were recorded on a JASCO P-1020 digital polarimeter (JASCO Corporation, Tokyo, Japan) and JASCO J-815 spectrophotometer (JASCO Corporation), respectively. A Varian 400 NMR instrument was used to record the ^1^H NMR and ^13^C NMR spectra with the chemical shifts shown as ppm referenced to the solvent residue of CDCl_3_ (*δ*_H_ 7.26 ppm and *δ*_C_ 77.0 ppm), DMSO-*d*_6_ (*δ*_H_ 2.50 ppm and *δ*_C_ 39.5 ppm), and CD_3_OD (*δ*_H_ 3.31 ppm and *δ*_C_ 49.0 ppm). A Bruker APEX II mass spectrometer equipped with an ESI ionization source (Bruker, Bremen, Germany) was used for acquiring high-resolution mass data. The HPLC system was composed of a Shimadzu LC-10AT*_VP_* series pump, a UV detector (Shimadzu, Milan, Italy), and an ODS column (5 µm, 250 × 10 mm, Inertsil ODS-3, GL Science Inc., Tokyo, Japan).

### 3.2. Animal Material

The collection of *S. glaucum* was performed off the coast of Jihui Fishing Port, Taitung county, Taiwan, in March 2013. A −20 °C freezer was used for specimen storage until extraction. Prof. Chang-Feng Dai performed the species identification, and a voucher specimen (JiH-201304) of this soft coral has been deposited in National Sun Yat-sen University.

### 3.3. Extraction and Isolation

The lyophilized samples of *S. glaucum* (195 g) were chopped and soaked (4 × 24 h) with EtOAc (4 × 2 L). After the solvent was evaporated, a residue (17.35 g) was obtained. The residue of the EtOAc layer was chromatographed by a silica gel column, using a stepwise gradient system composed of EtOAc/*n*-hexane (0:100 to 100:0, each four column volumes) and acetone/EtOAc (0:100 to 100:0, each four column volumes), and eventually washed by MeOH to afford 25 fractions according to TLC analysis. Fraction 19 was subjected to repeated column chromatography (CC) by Sephadex LH-20 (isocratic, acetone), C18 gel (MeOH-H_2_O, 75%), and silica gel (acetone-*n*-hexane, 10%) to obtain subfractions (SFr.) 19-1 and 19-2. In turn, SFr.19-1 was subjected to semipreparative HPLC (MeOH-H_2_O, 73%) to give compound **6** (23.4 mg), while compound **2** (1.3 mg) was obtained from SFr.19-2 using a CH_3_CN-H_2_O (53%) solvent system. Fraction 20 was subjected to an open column chromatography using Sephadex LH-20 (isocratic, acetone) and subsequently purified by C18 column chromatography (MeOH-H_2_O, 90%) to yield two subfractions (SFr. 20-1 and 20-2). Compounds **11** (2.7 mg) and **10** (2.0 mg) were purified from SFr. 20-1 using HPLC (MeOH-H_2_O, 79%). SFr.20-2 was fractionated into two subfractions (SFr. 20-2-1 and SFr. 20-2-2) using C18 CC (MeOH-H_2_O, 64%). Compounds **8** (1.7 mg) and **9** (6.9 mg) were yielded from SFr. 20-2-1 using HPLC (MeOH-H_2_O, 65%), and compounds **3** (2.7 mg) and **15** (2.1 mg) were obtained from SFr. 20-2-2 using eluent of CH_3_CN-H_2_O (33%). Four subfractions (SFr. 22-1–SFr.22-4) were obtained from fraction 22 by C18 CC (MeOH-H_2_O, 80%). Compounds **4** (4.9 mg) and **7** (4.7 mg) were purified from SFr.22-1 using HPLC (MeOH-H_2_O, 64%). Compounds **1** (2.2 mg), **12** (33.6 mg), and **13** (4.8 mg) were isolated from SFr. 22-2, SFr. 22-3, and SFr.22-4, respectively, using HPLC (MeOH-H_2_O: 71% for SFr. 22-2, 79% for SFr. 22-3, and 85% for SFr. 22-4). Fraction 23 was subjected to CC using a C18 column (MeOH-H_2_O, 80%) and subsequently by HPLC (MeOH-H_2_O, 66%) to give compounds **14** (3.6 mg) and **5** (1.4 mg).

3,4,8,16-Tetra-*epi*-lobocrasol (**1**): colorless oil; ${\left[\alpha \right]}_{\;D}^{25}$ –98 (*c* 1.00, CHCl_3_); ECD (MeOH) *λ*_max_ (Δε) 211 (−0.62), 233 (+1.79), 324 (−0.62); IR (KBr) ν_max_ 3419, 2925, 2855, 1698, 1651, 1455, 1380, 1332, 1246, 1080, and 1049 cm^−1^; ^13^C and ^1^H NMR data, see Table 1 and Table 2; ESIMS *m/z* 357 [M + Na]^+^; HRESIMS *m/z* 357.2034 [M + Na]^+^ (calculated (calcd.) for C_20_H_30_O_4_Na, 357.2036).

1,15β-Epoxy-deoxysarcophine (**2**): colorless oil; ${\left[\alpha \right]}_{\;D}^{25}$ −154 (*c* 0.63, CHCl_3_); IR (KBr) ν_max_ 2926, 2855, 1653, 1514, 1455, 1381, 1239, 1164, and 1039 cm^−1^; ^13^C and ^1^H NMR data, see Table 1 and Table 2; ESIMS *m/z* 341 [M + Na]^+^; HRESIMS *m/z* 341.2085 [M + Na]^+^ (calcd. for C_20_H_30_O_3_Na, 341.2087).

3,4-Dihydro-4α,7β,8α-trihydroxy-Δ^2^-sarcophine (**3**): colorless oil; ${\left[\alpha \right]}_{\;D}^{25}$ −84 (*c* 1.28, CHCl_3_); IR (KBr) ν_max_ 3444, 2965, 2929, 2857, 1747, 1668, 1638, 1455, 1373, 1317, 1260, 1127, 1062, and 1018 cm^−1^; ^13^C and ^1^H NMR data, see Table 1 and Table 2; ESIMS *m/z* 373 [M + Na]^+^; HRESIMS *m/z* 373.1986 [M + Na]^+^ (calcd. for C_20_H_30_O_5_Na, 373.1986).

*ent*-Sarcophyolide E (**4**): colorless oil; ${\left[\alpha \right]}_{\;D}^{25}$ +14 (*c* 1.70, CHCl_3_); ECD (MeOH) *λ*_max_ (Δε) 230 (+1.41), 254 (−0.40); ^13^C and ^1^H NMR data, see Table 1 and Table 2; ESIMS *m/z* 373 [M + Na]^+^; HRESIMS *m/z* 373.1984 [M + Na]^+^ (calcd. for C_20_H_30_O_5_Na, 373.1986).

16-Deacetyl-halicrasterol B (**5**): white powder; ${\left[\alpha \right]}_{\;D}^{25}$ −111 (*c* 0.78, CHCl_3_); IR (KBr) ν_max_ 3330, 2922, 2854,1652, 1598, 1455, 1374, and 1023 cm^−1^; ^13^C and ^1^H NMR data, see Table 3; ESIMS *m/z* 501 [M + Na]^+^; HRESIMS *m/z* 501.3552 [M + Na]^+^ (calcd. for C_29_H_50_O_5_Na, 501.3551).

Sarcophine (**6**): colorless oil; ${\left[\alpha \right]}_{\;D}^{25}$ +139 (*c* 4.98, CHCl_3_); lit. ${\left[\alpha \right]}_{\;D}^{25}$ +97 (*c* 0.01, CHCl_3_); ECD (MeOH) *λ*_max_ (Δε) 229 (+1.22), 248 (−1.58); MS, ^1^H and ^13^C NMR data were found to be in full agreement with literature data [11].

Lobocrasol (**7**): colorless oil; ${\left[\alpha \right]}_{\;D}^{25}$ −212 (*c* 0.05, CHCl_3_); lit. ${\left[\alpha \right]}_{\;D}^{25}$ −186 (*c* 0.10, CHCl_3_); ECD (MeOH) *λ*_max_ (Δε) 215 (+0.57), 240 (−1.66), 325 (+0.81); MS, ^1^H and ^13^C NMR data were found to be in full agreement with literature data [12].

3,4-Dihydro-4α-hydroxy-Δ^2^-sarcophine (**8**): colorless oil; ${\left[\alpha \right]}_{\;D}^{25}$ −17 (*c* 0.50, CHCl_3_); lit. ${\left[\alpha \right]}_{\;D}^{25}$ −5 (*c* 0.10, CHCl_3_); MS, ^1^H and ^13^C NMR data were found to be in full agreement with literature data [11,13].

3,4-Dihydro-4β-hydroxy-Δ^2^-sarcophine (**9**): colorless oil; ${\left[\alpha \right]}_{\;D}^{25}$ −1 (*c* 1.53, CHCl_3_); lit. ${\left[\alpha \right]}_{\;D}^{25}$ −1 (*c* 0.10, CHCl_3_); MS, ^1^H and ^13^C NMR data were found to be in full agreement with literature data [11,13].

Srassumol A (**10**): colorless oil; ${\left[\alpha \right]}_{\;D}^{25}$ −30 (*c* 0.49, CHCl_3_); lit. ${\left[\alpha \right]}_{\;D}^{25}$ −20 (*c* 0.10, CHCl_3_); MS, ^1^H and ^13^C NMR data were found to be in full agreement with literature data [14].

Klyflaccicembranol F (**11**): colorless oil; ${\left[\alpha \right]}_{\;D}^{25}$ −76 (*c* 1.28, CHCl_3_); lit. ${\left[\alpha \right]}_{\;D}^{25}$ −32 (*c* 0.60, CHCl_3_); MS, ^1^H and ^13^C NMR data were found to be in full agreement with literature data [15].

Sarcomilasterol (**12**): amorphous solid; ${\left[\alpha \right]}_{\;D}^{25}$ +30 (*c* 0.67, CHCl_3_); lit. ${\left[\alpha \right]}_{\;D}^{20}$ +5 (*c* 0.08, CHCl_3_); MS, ^1^H and ^13^C NMR data were found to be in full agreement with literature data [16].

Sarcoaldesterol B (**13**): amorphous solid; ${\left[\alpha \right]}_{\;D}^{25}$ −21 (*c* 1.50, MeOH); lit. ${\left[\alpha \right]}_{\;D}^{25}$ −20 (*c* 0.23, MeOH); MS, ^1^H and ^13^C NMR data were found to be in full agreement with literature data [17].

Sarglaucsterol (**14**): amorphous solid; ${\left[\alpha \right]}_{\;D}^{25}$ −15 (*c* 0.45, MeOH); lit. ${\left[\alpha \right]}_{\;D}^{20}$ −25 (*c* 0.11, MeOH); MS, ^1^H and ^13^C NMR data were found to be in full agreement with literature data [18].

Loliolide (**15**): colorless oil; ${\left[\alpha \right]}_{\;D}^{25}$ −11 (*c* 0.73, CHCl_3_); lit. ${\left[\alpha \right]}_{\;D}$ −92 (*c* 1.10, CHCl_3_); MS, ^1^H and ^13^C NMR data were found to be in full agreement with literature data [19].

### 3.4. Cytotoxicity Assay

HepG2, MDA-MB-231, A-549, MOLT-4, SUP-T1, and U-937 cells were obtained from the American Type Culture Collection (ATCC; Manassas, VA, USA). The MTT assays were performed as previous reported [25]. After a 15 h culture for the above cancer cell lines, the isolated compounds prepared in different concentrations of DMSO were added for an additional 72 h culture [25]. The cytotoxic potential of the isolated compounds was evaluated by means of a MTT [3-(4,5-dimethylthiazole-2-yl)-2,5-diphenyltetrazolium bromide] cell proliferation assay and the absorbance was measured using an ELISA reader and monitored at 570 and 620 nm, which allowed the calculation of IC_50_ values.

## 4. Conclusions

Our present study discovered four new diterpenoids **1**–**4** and a new steroid 5 as well as ten known compounds **6–15**. The 3,4,8,16-tetra-*epi*-lobocrasol (**1**) represent the second example of lobocrasol-type diterpenoid, while the Δ^11^ double in lobocrasol (**7**) was revised as *E* geometry in this study. It is interesting that, similar to sarcophine and *ent*-sarcophine [26], sarcophyolide E and *ent*-sarcophyolide E (**4**) were also isolated from the same genus.

## Figures and Tables

**Table 1:** ^13^C NMR spectroscopic data of compounds **1**‒**3** (100 MHz, CDCl_3_) and **4** (100 MHz, DMSO-*d*_6_).

No.	1	2	3	4
1	142.8 (C)	71.9 (C)	152.2 (C)	165.3 (C)
2	204.3 (C)	76.6 (CH)	148.2 (C)	79.6 (CH)
3	59.4 (CH)	122.4 (CH)	116.5 (CH)	119.5 (CH)
4	83.2 (C)	140.0 (C)	74.2 (C)	143.8 (C)
5	32.4 (CH_2_)	37.9 (CH_2_)	38.8 (CH_2_)	35.7 (CH_2_)
6	25.0 (CH_2_)	25.4 (CH_2_)	26.9 (CH_2_)	24.2 (CH_2_)
7	83.8 (CH)	61.9 (CH)	74.6 (CH)	83.4 (CH)
8	73.6 (C)	59.8 (C)	74.9 (C)	68.3 (C)
9	34.0 (CH_2_)	40.2 (CH_2_)	38.0 (CH_2_)	40.3 (CH_2_)
10	21.5 (CH_2_)	23.7 (CH_2_)	22.1 (CH_2_)	23.0 (CH_2_)
11	128.4 (CH)	123.9 (CH)	128.5 (CH)	80.0 (CH)
12	133.3 (C)	136.1 (C)	131.4 (C)	71.3 (C)
13	39.1 (CH_2_)	35.2 (CH_2_)	38.3 (CH_2_)	36.8 (CH_2_)
14	20.0 (CH_2_)	27.5 (CH_2_)	22.3 (CH_2_)	20.1 (CH_2_)
15	167.0 (C)	67.4 (C)	123.7 (C)	121.2 (C)
16	75.9 (CH)	69.4 (CH_2_)	170.3 (C)	174.5 (C)
17	13.2 (CH_3_)	12.1 (CH_3_)	8.8 (CH_3_)	8.5 (CH_3_)
18	25.2 (CH_3_)	15.6 (CH_3_)	29.5 (CH_3_)	16.2 (CH_3_)
19	25.8 (CH_3_)	16.7 (CH_3_)	25.5 (CH_3_)	20.0 (CH_3_)
20	15.3 (CH_3_)	14.9 (CH_3_)	15.9 (CH_3_)	23.4 (CH_3_)

**Table 2:** ^1^H NMR spectroscopic data of compounds **1**‒**3** (400 MHz, CDCl_3_) and **4** (400 MHz, DMSO-*d*_6_).

No.	1	2	3	4
2	-	4.87 d (11.2)	-	5.53 d (10.4)
3	2.16 br s	5.26 dd (10.8, 0.8)	5.21 s	4.99 d (10.4)
5	3.04 q (10.8)	2.34 m	2.09 m	2.18 m
	1.67 m	-	1.78 m	-
6	2.00 dd (9.6, 2.4)	1.92 m	1.54 m	1.97 m
	1.54 m	1.63 m	1.51 m	1.42 m
7	3.80 dd (9.2, 5.6)	2.65 t (4.0)	3.52 dd (9.2, 3.2)	3.01 dd (10.0, 2.8)
9	1.50 m	2.13 m	1.64 m	1.73 m
	1.25 m	0.93 m	1.47 m	1.49 m
10	2.05 m	2.27 m	2.17 m	1.62 m
	1.90 m	1.87 m	1.86 m	1.34 m
11	5.15 t (7.6)	5.11 dd (9.6, 5.6)	4.99 t (6.8)	3.13 d (10.4)
13	2.28 br d (13.2)	2.22 m	2.28 m	1.70 m
	1.84 m	1.95 m	-	1.49 m
14	2.61 td (13.2, 2.1)	1.69 m	2.56 m	2.45 m
	2.12 m	-	-	1.91 m
16	4.90 br s	3.89 d (10.4)	-	-
	-	3.75 d (10.4)	-	-
17	2.08 s	1.43 s	1.94 s	1.73 s
18	1.35 s	1.84 s	1.40 s	1.77 s
19	1.03 s	1.27 s	1.61 s	0.98 s
20	1.69 s	1.57 s	1.67 s	0.98 s

**Table 3:** ^1^H and ^13^C NMR spectroscopic data of compound **5**.

No.	*δ*_H_ (*J* in Hz) ^a^	*δ*_C_ (mult.) ^b^	No.	*δ*_H_ (*J* in Hz) ^a^	*δ*_C_ (mult.) ^b^
1	1.99 m	35.2 (CH_2_)	15	1.62 m	36.5 (CH_2_)
	1.54 m	-	-	1.45 m	-
2	1.75 m	32.0 (CH_2_)	16	4.62 d (5.2)	73.0 (CH)
	1.51 m	-	17	-	148.2 (C)
3	4.00 m	68.2 (CH)	18	0.91 s	18.8 (CH_3_)
4	2.09 dd (13.2, 11.6)	42.0 (CH_2_)	19	1.28 s	17.5 (CH_3_)
	1.54 m	-	20	-	134.2 (C)
5	-	77.4 (C)	21	1.77 s	17.5 (CH_3_)
6	3.47 br s	76.6 (CH)	22	2.41 dd (13.2, 10.8)	43.0 (CH_2_)
7	1.84 m	35.3 (CH_2_)	-	1.91 m	-
8	1.88 m	29.1 (CH)	23	1.86 m	34.0 (CH)
9	1.57 m	53.3 (CH)	24	1.09 m	45.9 (CH)
10	-	41.1 (C)	25	1.64 m	31.3 (CH)
11	3.88 td (10.4, 5.2)	69.6 (CH)	26	0.87 d (6.8)	19.6 (CH_3_)
12	2.62 dd (12.0, 5.2)	50.2 (CH_2_)	27	0.93 d (6.8)	22.0 (CH_3_)
	1.58 m	-	28	0.82 d (6.8)	11.9 (CH_3_)
13	-	46.0 (C)	29	0.72 d (6.8)	13.9 (CH_3_)
14	1.78 m	52.5 (CH)	-	-	-

^a^ Spectra recorded at 400 MHz in CD_3_OD; ^b^ spectra recorded at 100 MHz in CD_3_OD.

## Supplementary Materials

The ^1^H and ^13^C NMR spectra of compounds **1**–**5** and **7** (Figures S1–S6), and partial NOESY spectrum of **7** (Figure S6-3) are available online at http://www.mdpi.com/1660-3397/15/7/202/s1.
