# Supplementary material for: Isoprenoids from the Soft Coral Sarcophyton glaucum
Source: Mar Drugs. 2017 Jun 27;15(7):202. doi: 10.3390/md15070202 (PMC5532644; doi:10.3390/md15070202)
Supplement: Supplementary file 1 [file marinedrugs-15-00202-s001.pdf]

Isoprenoids from the Soft Coral *Sarcophyton glaucum*

Chih-Hua Chao[1,2,†], Wen-Liang Li [3,†], Chiung-Yao Huang [3], Atallah F. Ahmed [4], Chang-Feng Dai [5], Yang-Chang Wu [6,7,8], Mei-Chin Lu [9,10], Chih-Chuang Liaw [3] and Jyh-Horng Sheu [3,6,11,12,*]

[1] School of Pharmacy, China Medical University, Taichung 404, Taiwan; chaochihhua@hotmail.com

[2] Chinese Medicine Research and Development Center, China Medical University Hospital, Taichung 404, Taiwan

[3] Department of Marine Biotechnology and Resources, National Sun Yat-sen University, Kaohsiung 804, Taiwan; adj456@hotmail.com (W.-L.L.); huangcy@mail.nsysu.edu.tw (C.-Y.H.); ccliaw@mail.nsysu.edu.tw (C.-C.L.)

[4] Department of Pharmacognosy, College of Pharmacy, King Saud University, Riaydh 11451, Saudi Arabia; afahmed@ksu.edu.sa

[5] Institute of Oceanography, National Taiwan University, Taipei 112, Taiwan; corallab@ntu.edu.tw

[6] Graduate Institute of Natural Products, Kaohsiung Medical University, Kaohsiung 807, Taiwan; yachwu@mail.cmu.edu.tw

[7] Research Center for Natural Products & Drug Development, Kaohsiung Medical University, Kaohsiung 807, Taiwan

[8] Department of Medical Research, Kaohsiung Medical University Hospital, Kaohsiung 807, Taiwan

[9] Graduate Institute of Marine Biotechnology, National Dong Hwa University, Pingtung 944, Taiwan; jinx6609@nmmba.gov.tw

[10] National Museum of Marine Biology & Aquarium, Pingtung 944, Taiwan; pjsung@nmmba.gov.tw

[11] Department of Medical Research, China Medical University Hospital, China Medical University, Taichung 404, Taiwan

[12] Frontier Center for Ocean Science and Technology, National Sun Yat-sen University, Kaohsiung 804, Taiwan

*Correspondence: sheu@mail.nsysu.edu.tw; Tel: +886-7-5252000 (ext. 5030); Fax: +886-7-5255020

† These authors contributed equally to this work

For compound 1:
Figure S1-1. ^{1}H NMR spectrum (400 MHz) of compound **1** in $CDCl_3$.
Figure S1-2. ^{13}C NMR spectrum (100 MHz) of compound **1** in $CDCl_3$.
Figure S1-3. NOESY spectrum of compound **1** in $CDCl_{3.}$

For compound 2:
Figure S2-1. ^{1}H NMR spectrum (400 MHz) of compound **2** in $CDCl_3$.
Figure S2-2. ^{13}C NMR spectrum (100 MHz) of compound **2** in $CDCl_3$.

For compound 3:
Figure S3-1. ^{1}H NMR spectrum (400 MHz) of compound **3** in $CDCl_3$.
Figure S3-2. ^{13}C NMR spectrum (100 MHz) of compound **3** in $CDCl_3$.

For compound 4:
Figure S4-1. ^{1}H NMR spectrum (400 MHz) of compound **4** in DMSO.
Figure S4-2. ^{13}C NMR spectrum (100 MHz) of compound **4** in DMSO.

For compound 5:
Figure S5-1. ^{1}H NMR spectrum (400 MHz) of compound **5** in CD_3OD.
Figure S5-2. ^{13}C NMR spectrum (100 MHz) of compound **5** in CD_3OD.

For compound 7:
Figure S6-1. ^{1}H NMR spectrum (400 MHz) of compound **7** in $CDCl_3$.
Figure S6-2. ^{13}C NMR spectrum (100 MHz) of compound **7** in $CDCl_3$.
Figure S6-3. Partial NOESY spectrum of compound **7** in $CDCl_3$.

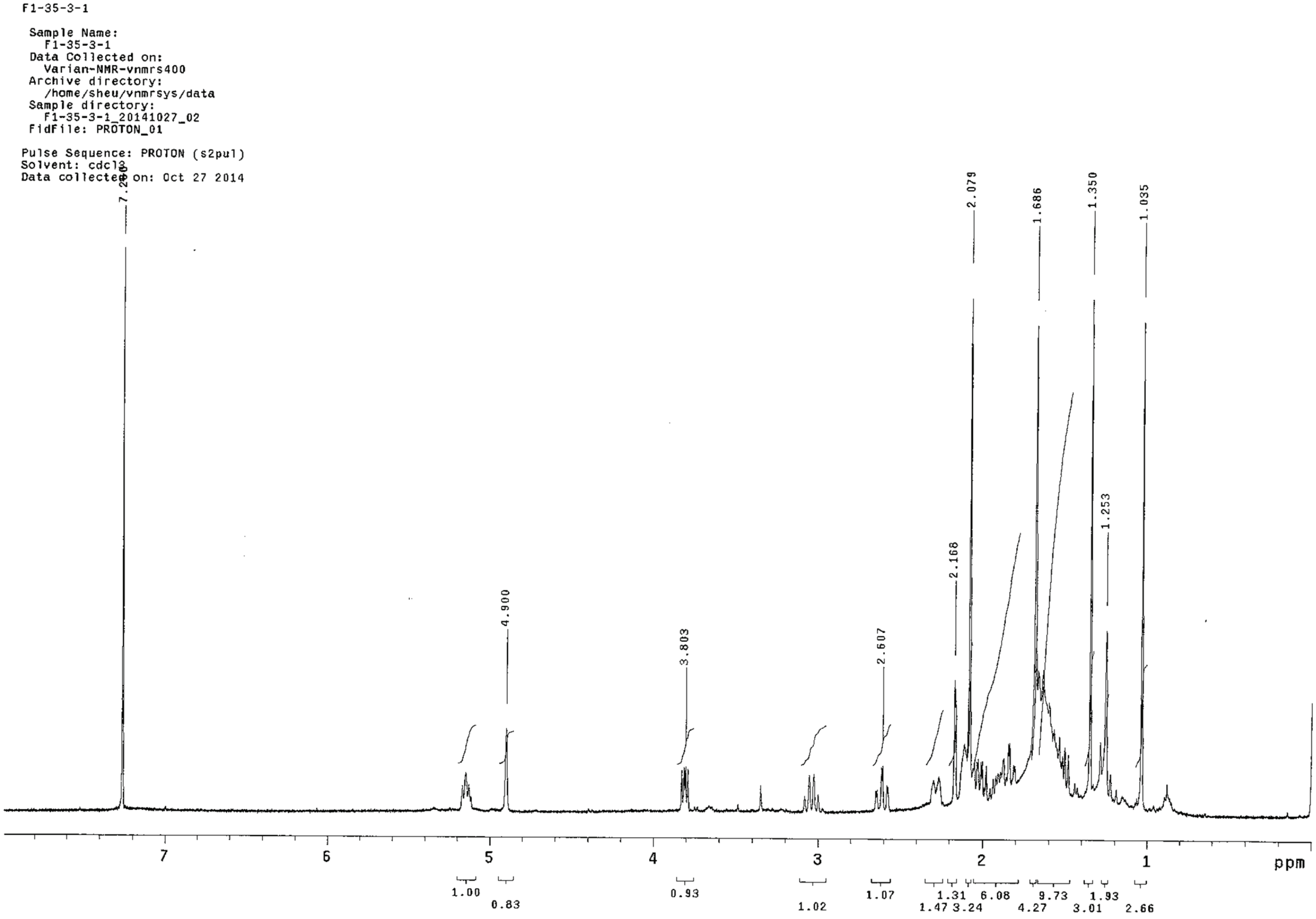

Figure S1-1. ^{1}H NMR spectrum (400 MHz) of compound **1** in $CDCl_3$.

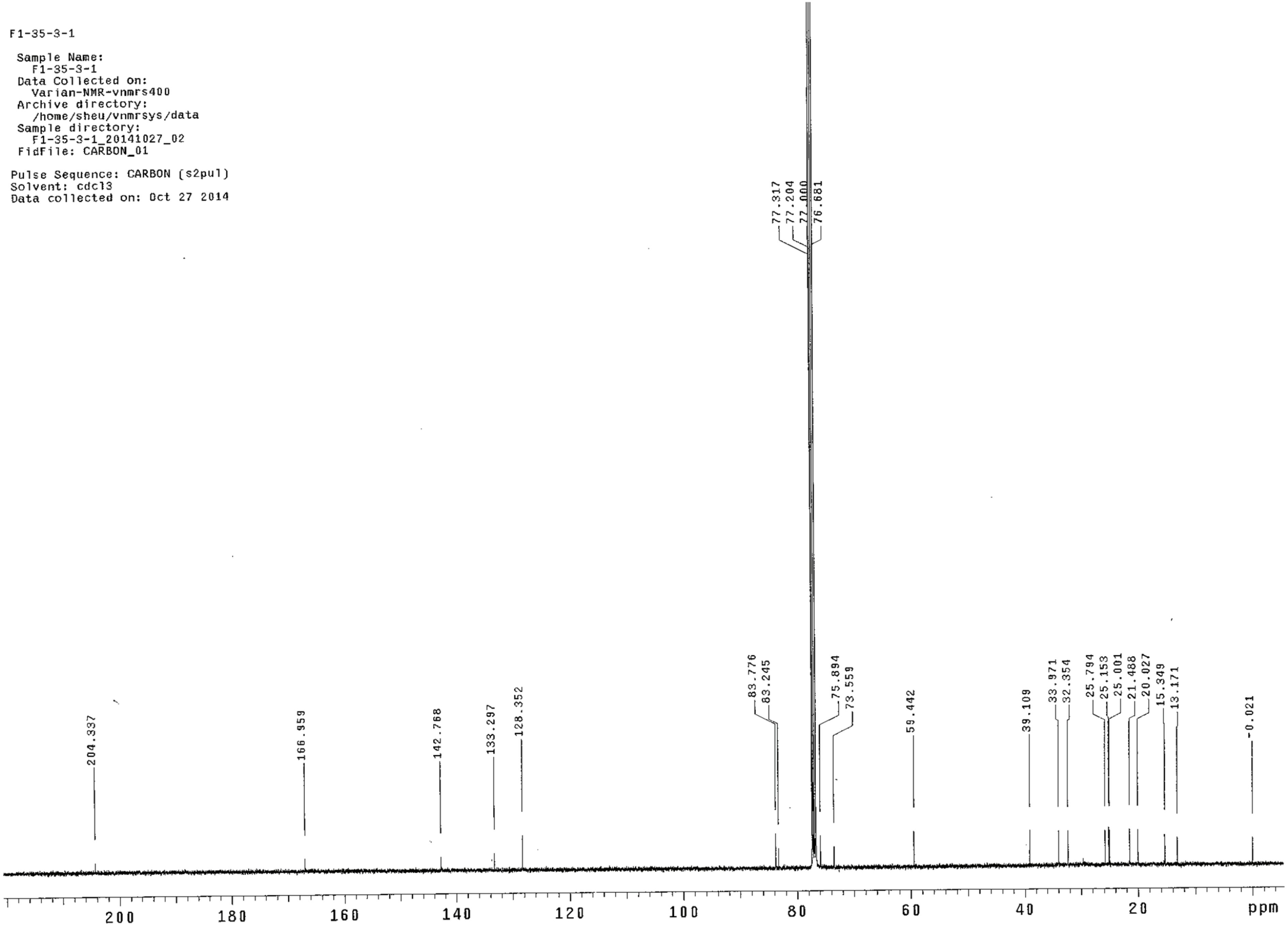

Figure S1-2. ^{13}C NMR spectrum (100 MHz) of compound **1** in $CDCl_3$.

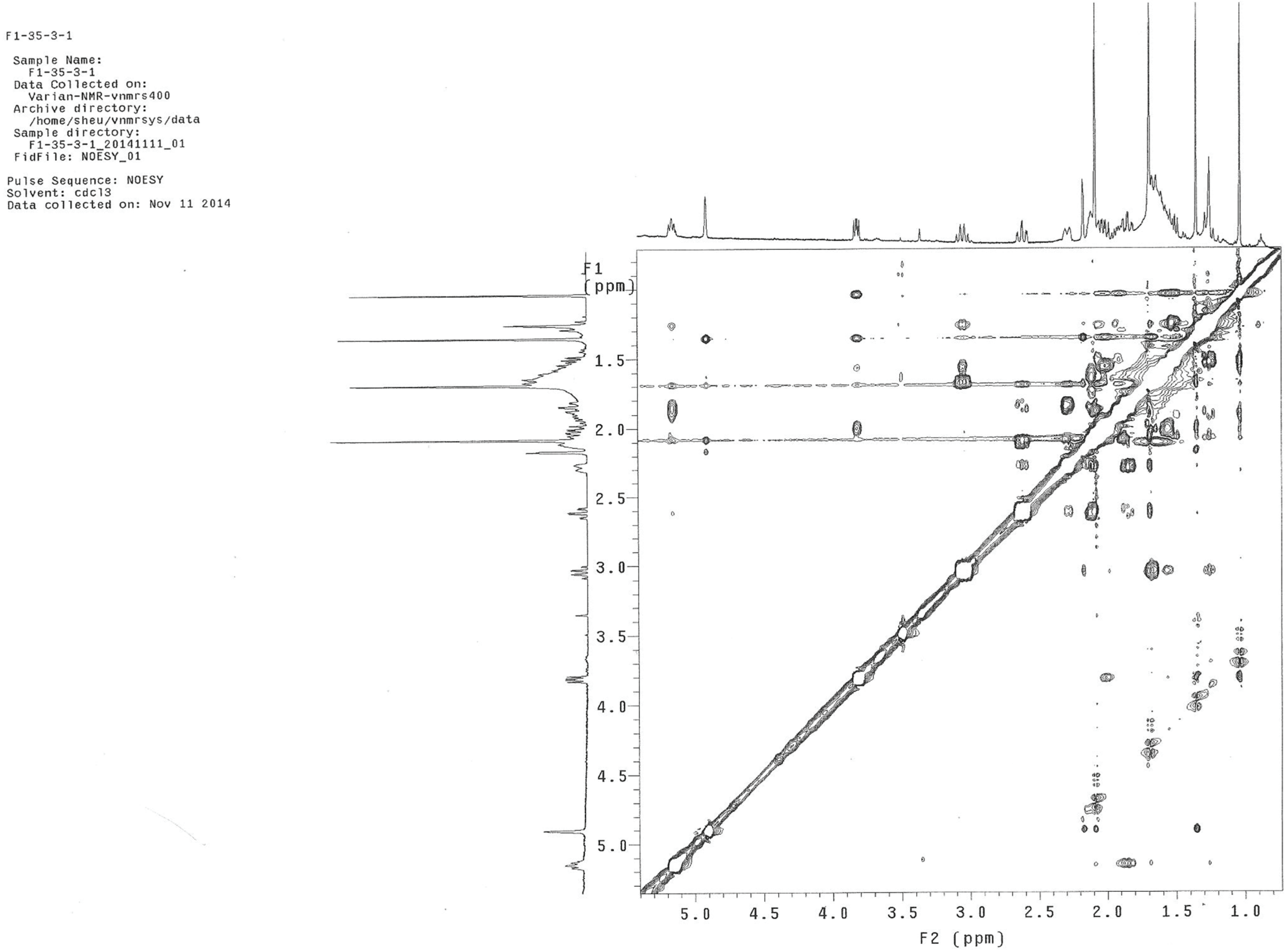

Figure S1-3. NOESY spectrum of compound **1** in $CDCl_3$.

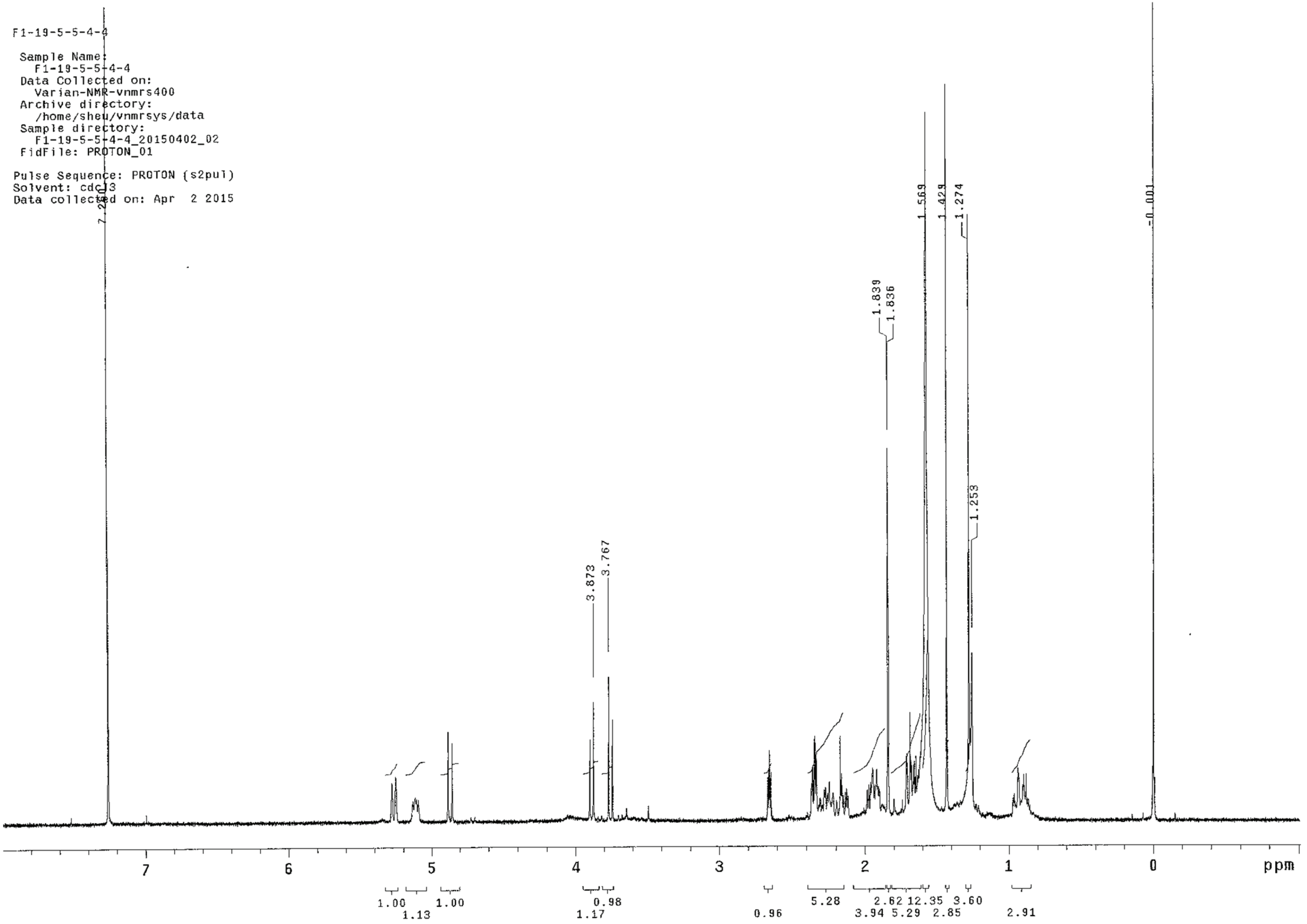

Figure S2-1. ^{1}H NMR spectrum (400 MHz) of compound **2** in $CDCl_3$

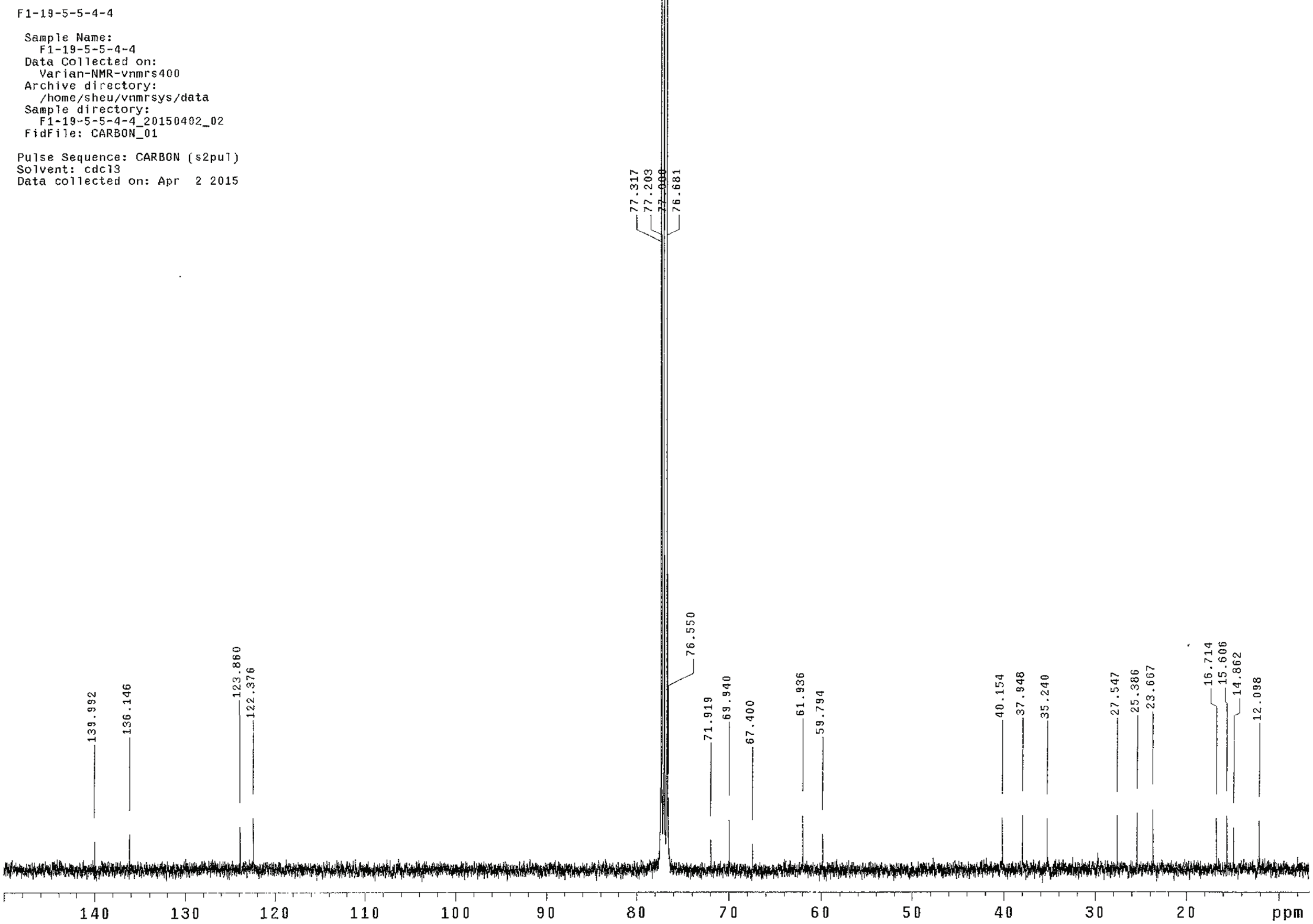

Figure S2-2. ^{13}C NMR spectrum (100 MHz) of compound **2** in $CDCl_3$.

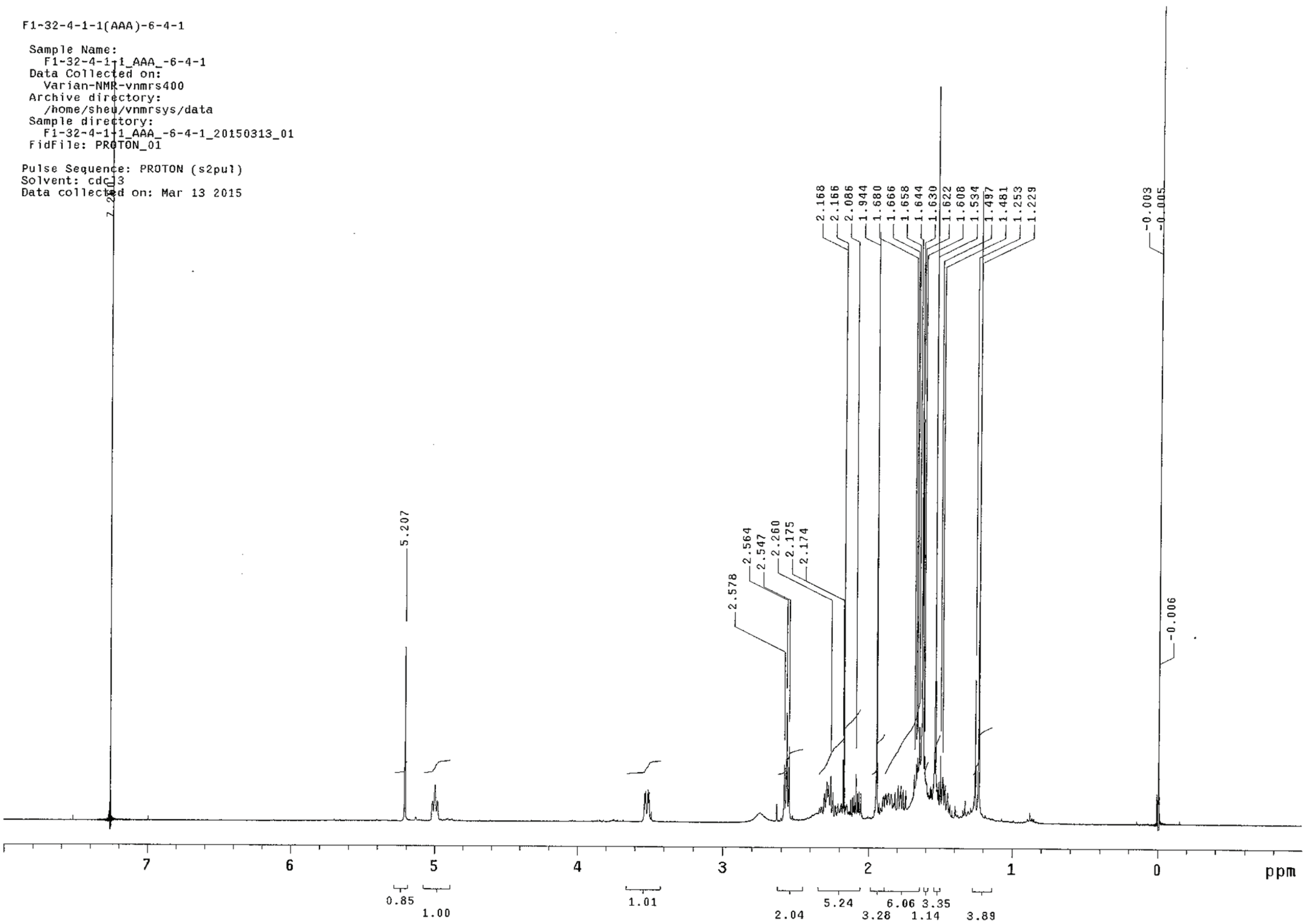

Figure S3-1. ^{1}H NMR spectrum (400 MHz) of compound **3** in $CDCl_3$.

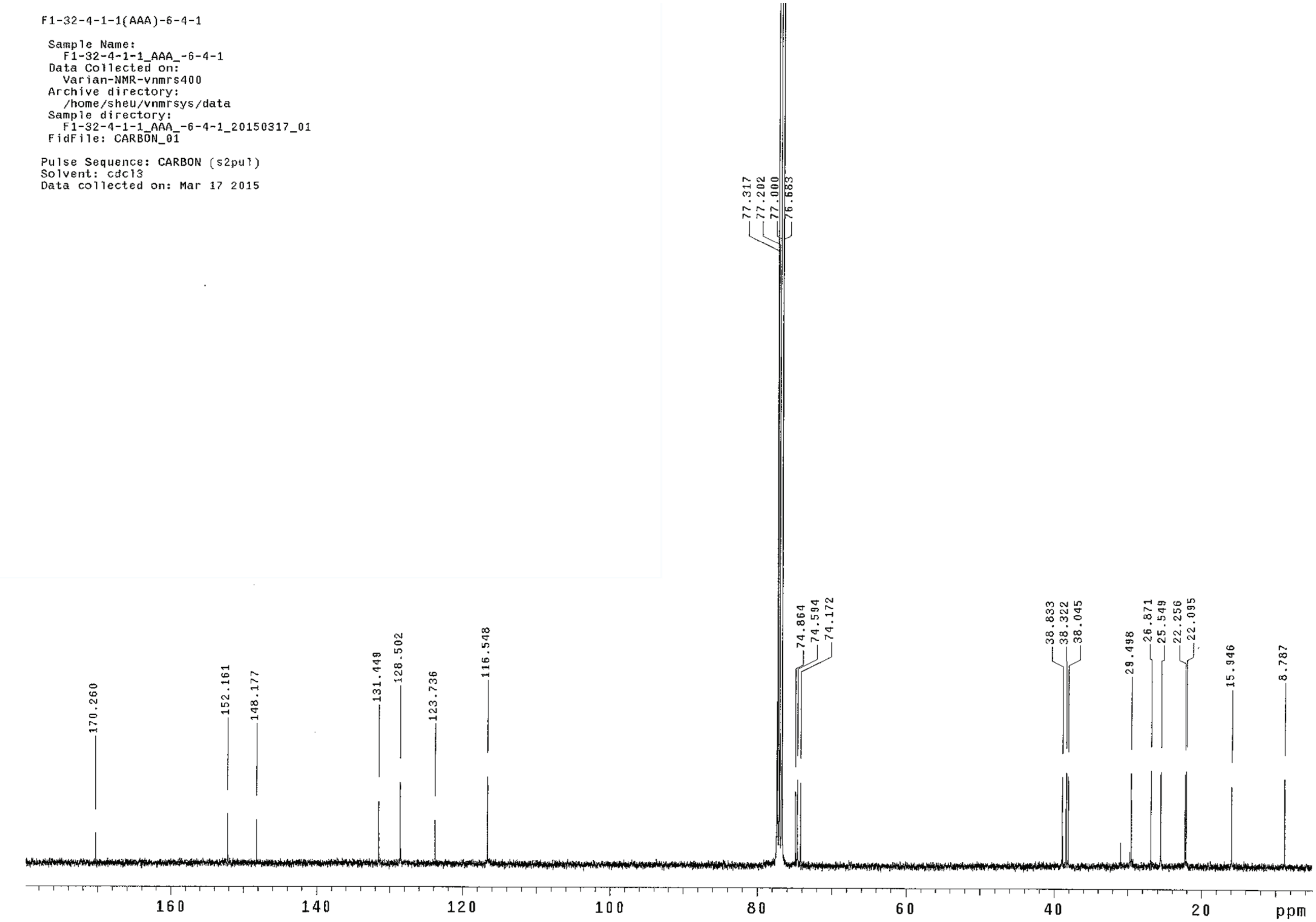

Figure S3-2. ^{13}C NMR spectrum (100 MHz) of compound **3** in $CDCl_3$

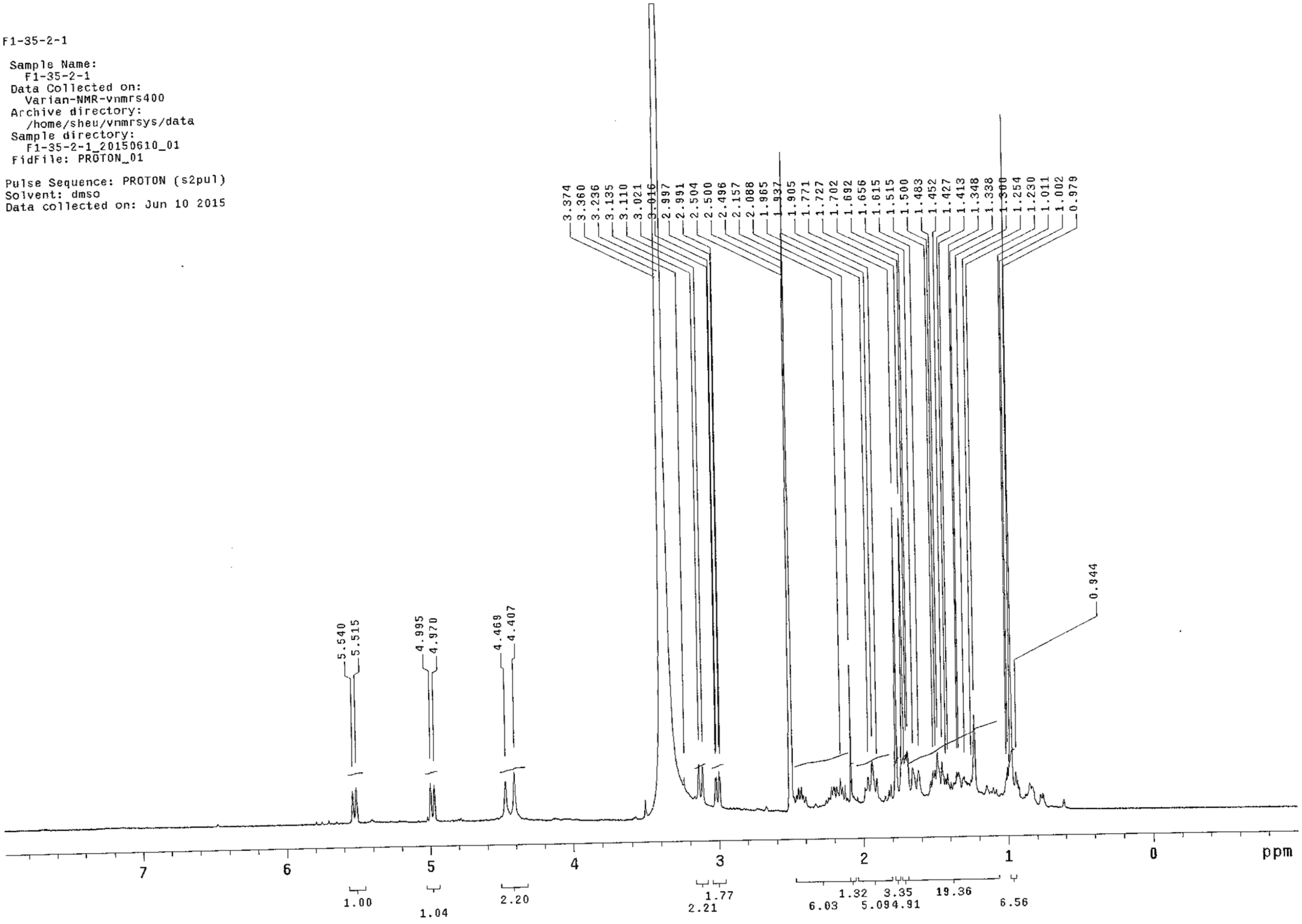

Figure S4-1. ^{1}H NMR spectrum (400 MHz) of compound **4** in DMSO.

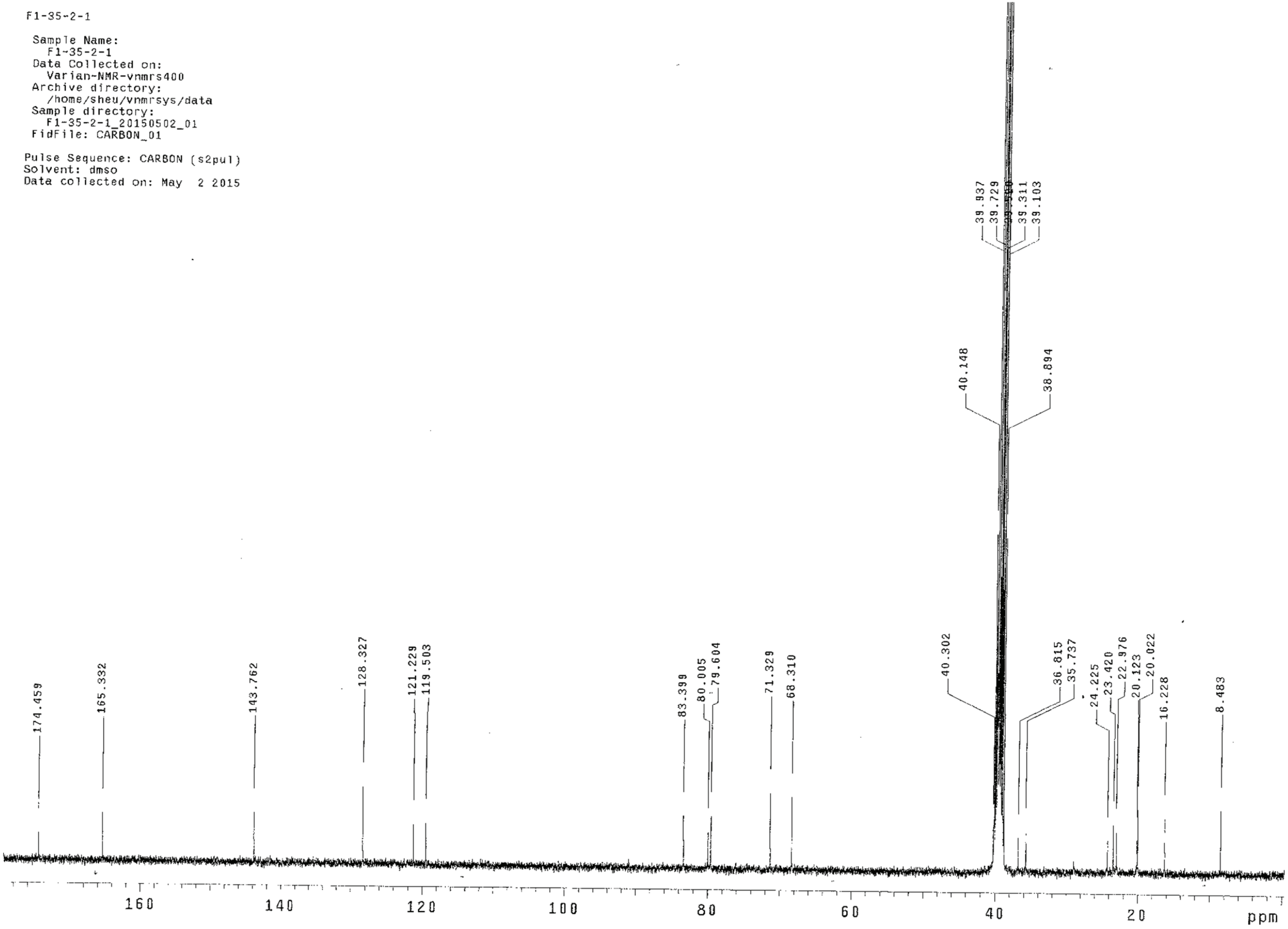

Figure S4-2. ^{13}C NMR spectrum (100 MHz) of compound **4** in DMSO.

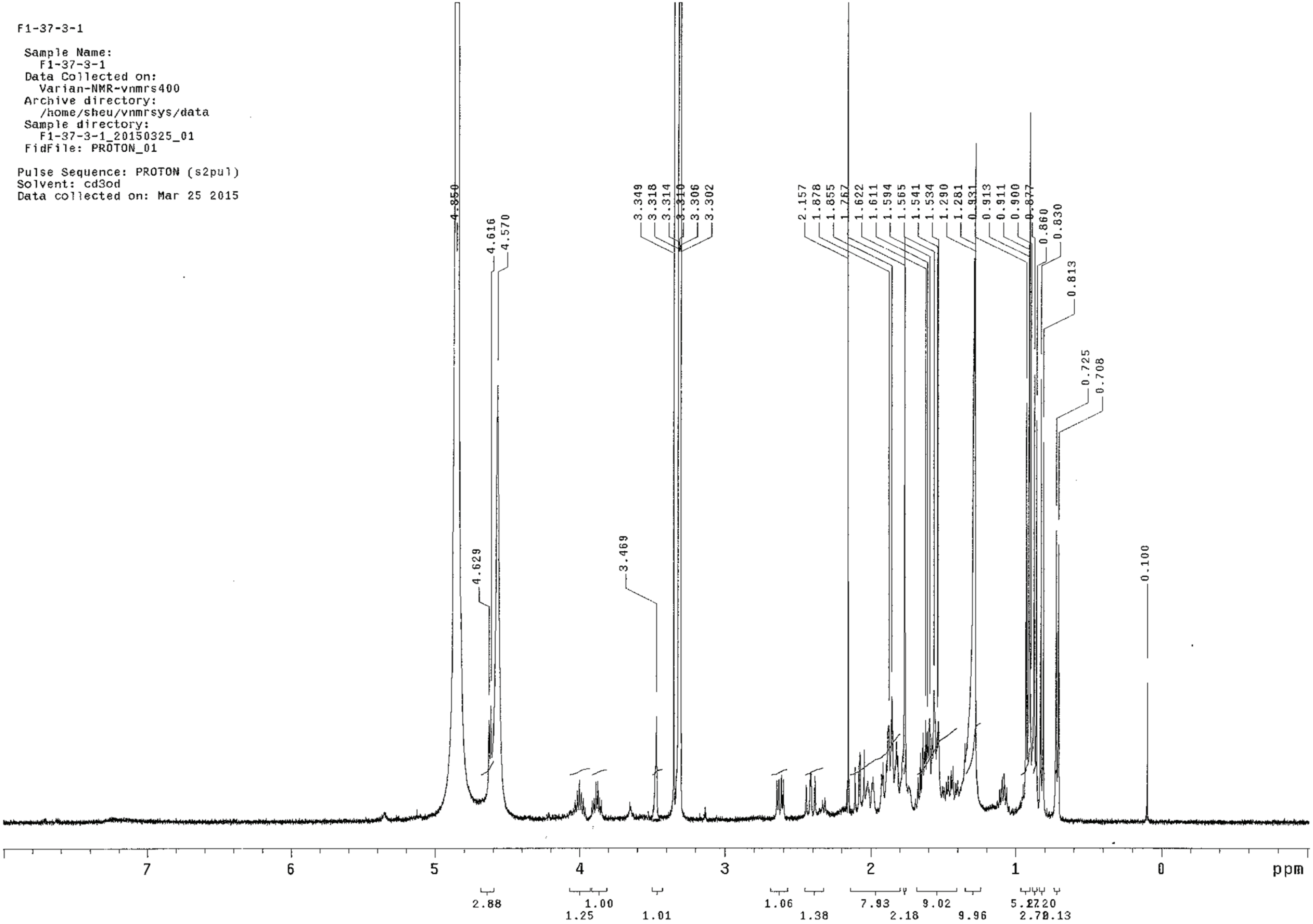

Figure S5-1. ^{1}H NMR spectrum (400 MHz) of compound **5** in CD_3OD.

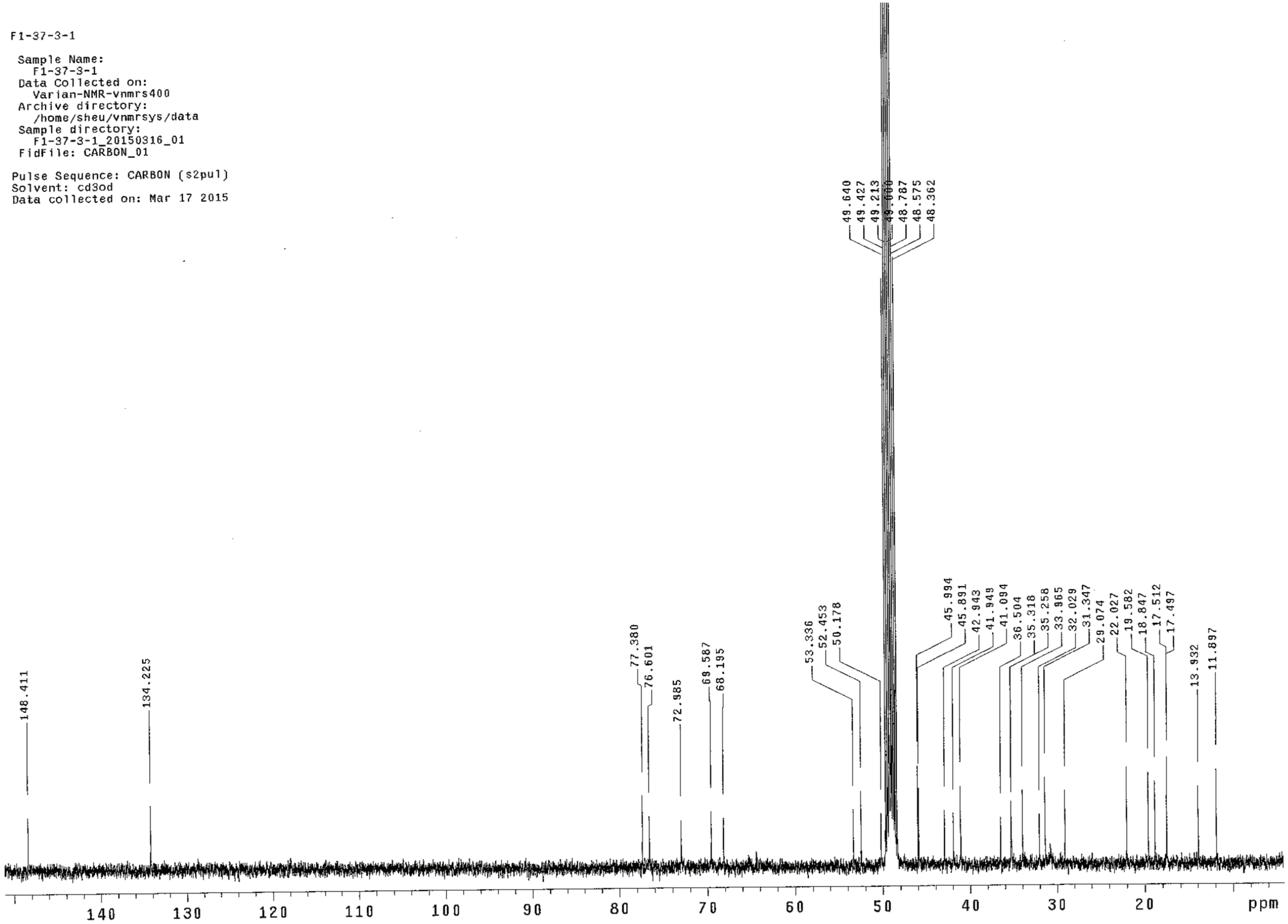

Figure S5-2. ^{13}C NMR spectrum (100 MHz) of compound **5** in CD_3OD.

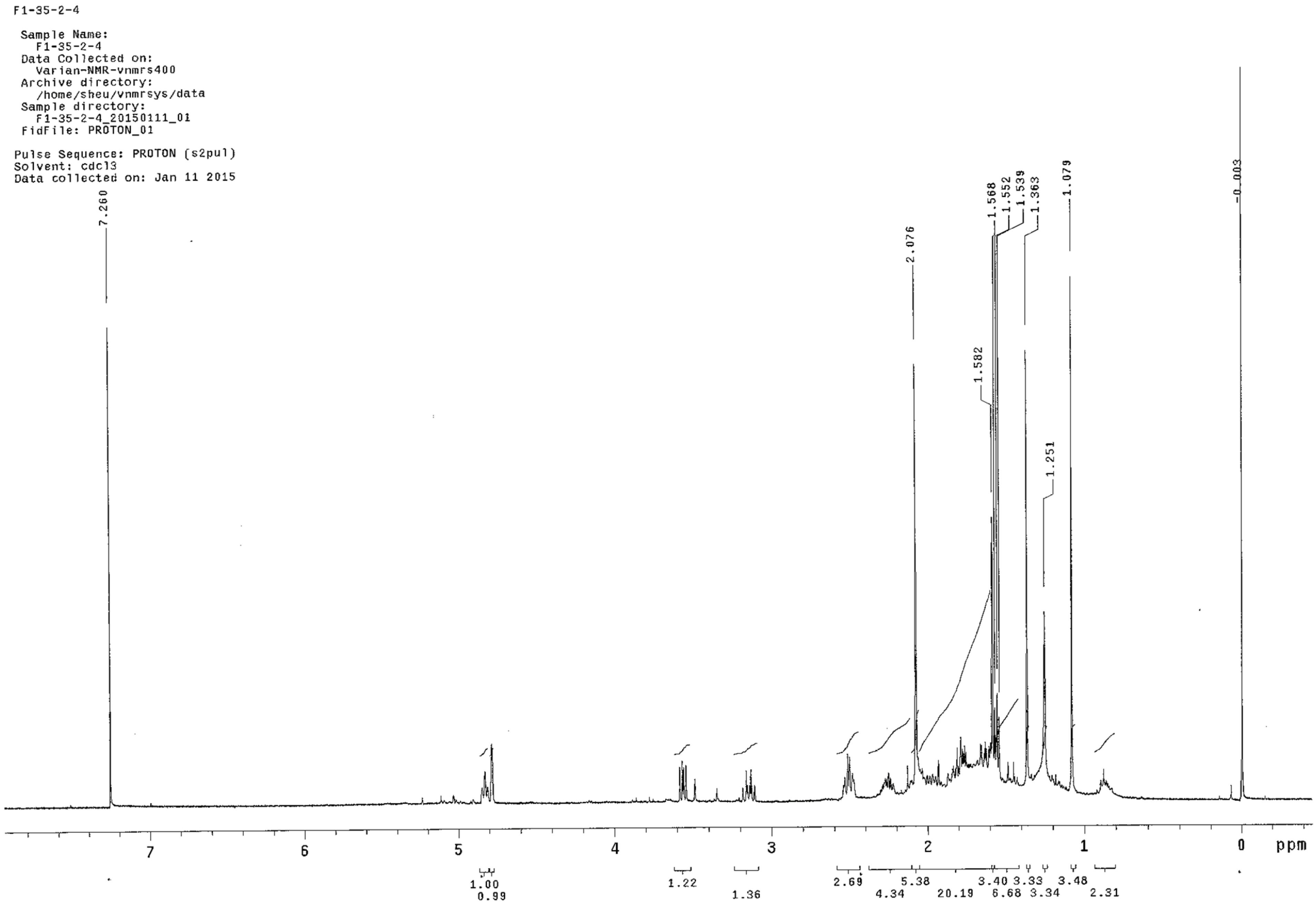

Figure S6-1. ^{1}H NMR spectrum (400 MHz) of compound **7** in $CDCl_3$.

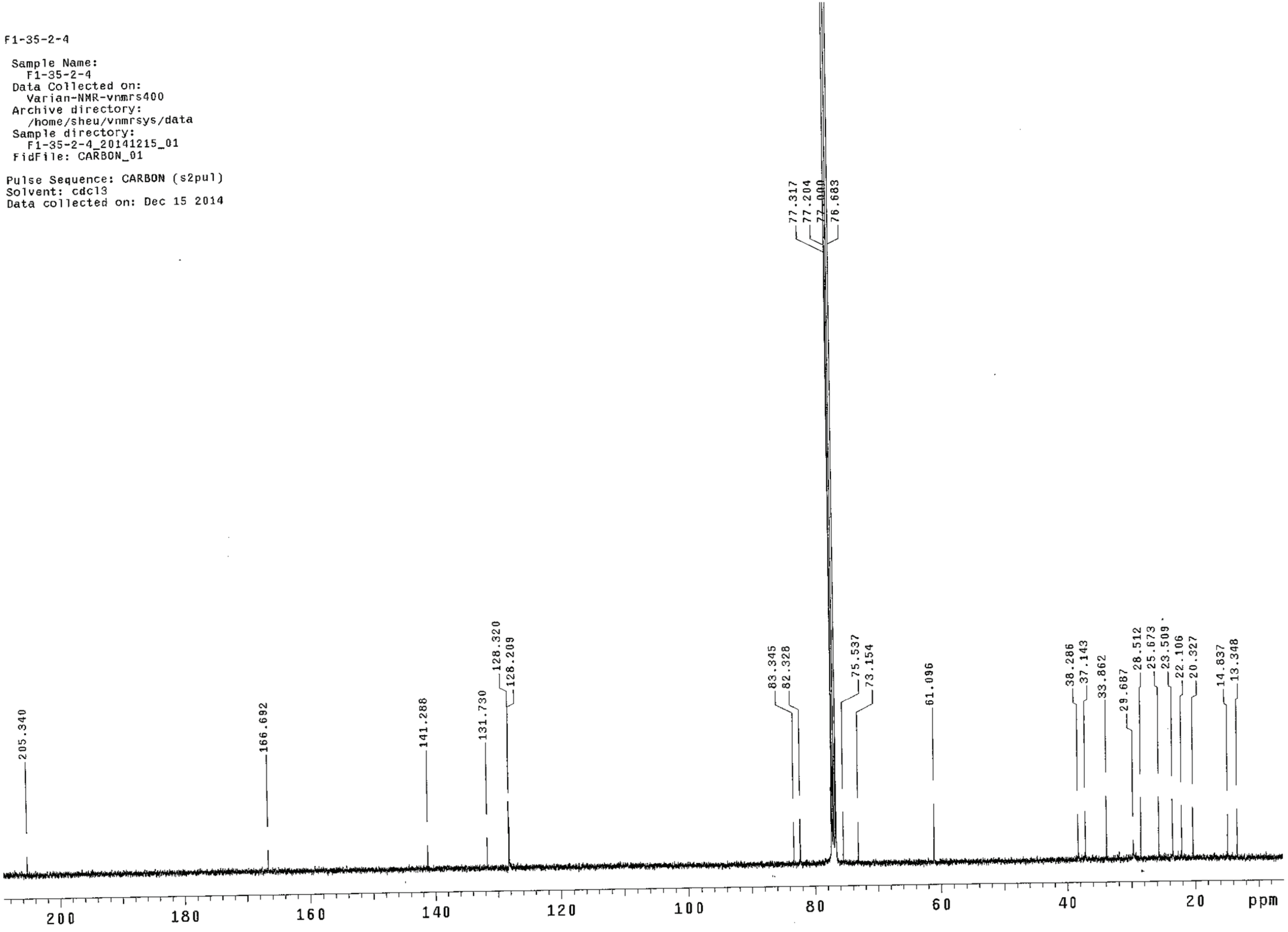

Figure S6-2. ^{13}C NMR spectrum (100 MHz) of compound **7** in $CDCl_3$.

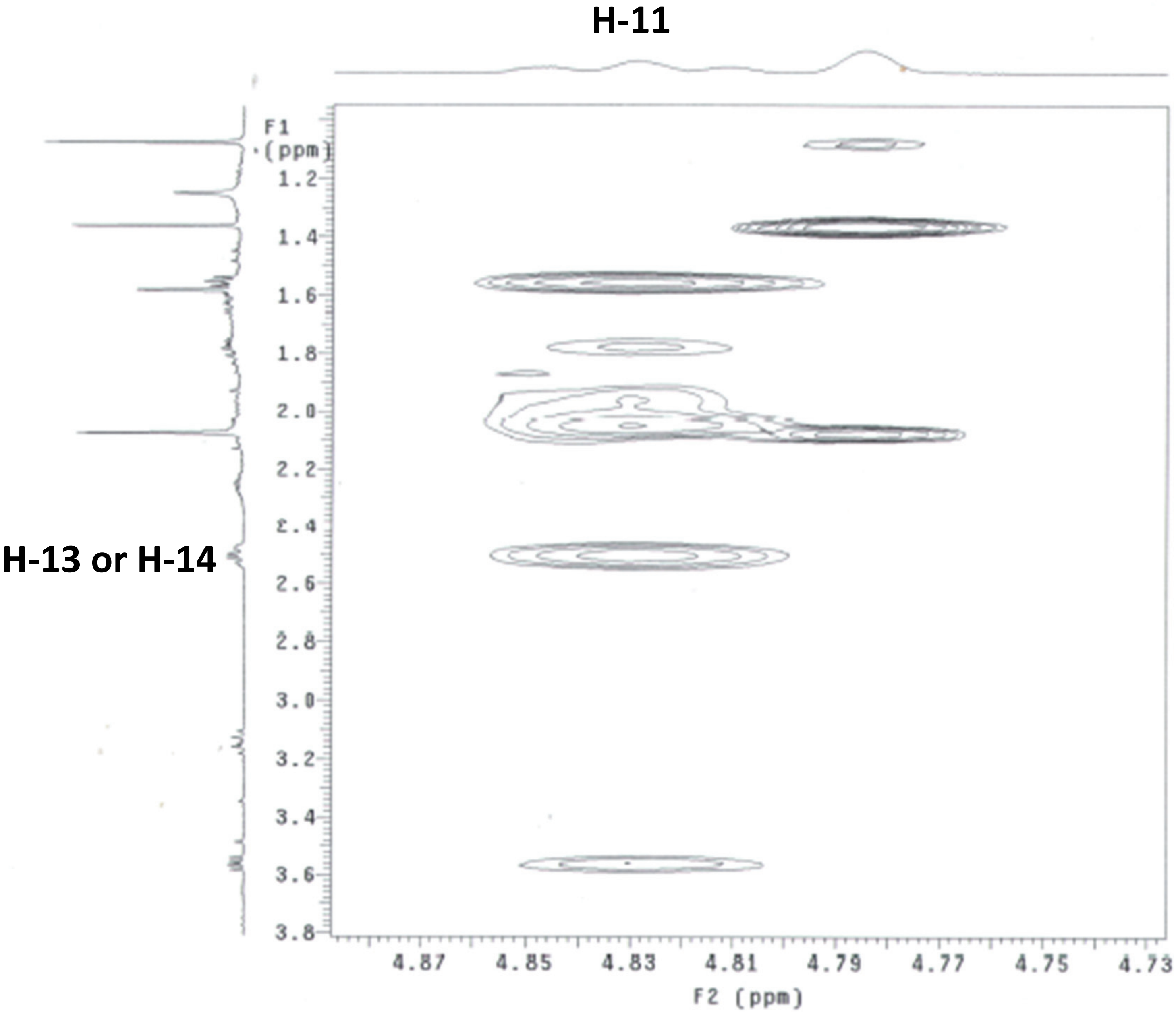

Figure S6-3. Partial NOESY spectrum of compound **7** in $CDCl_3$.
